# Comparison of meat quality and glycolysis potential of two hybrid pigs in three-way hybrid model

**DOI:** 10.3389/fvets.2023.1136485

**Published:** 2023-02-17

**Authors:** Yongxiang Li, Yang He, Jinming Ran, Ying Huang, Xian Li, Hengxin Jiang, Xueyan Li, Yangsu Pan, Sumei Zhao, Chunlian Song, Hongbin Pan, Hong Hu

**Affiliations:** ^1^Yunnan Provincial Key Laboratory of Animal Nutrition and Feed Science, Faculty of Animal Science and Technology, Yunnan Agricultural University, Kunming, China; ^2^College of Modern Agriculture, Dazhou Vocational and Technical College, Dazhou, China; ^3^College of Veterinary Medicine, Yunnan Agricultural University, Kunming, China

**Keywords:** Saba pig, meat quality, glycolysis potential, muscle fibers, three-way crossbred

## Abstract

With the improvement of consumers' requirements for pork quality, the method of crossbreeding with excellent local pig breeds to improve meat quality is popular. Saba pig has high reproduction rate, good meat quality and high utilization rate of roughage, but its excellent characteristics have not been fully developed and utilized. To promote the development and utilization of Saba pigs and production of high-quality pork, the meat quality traits and glycolysis potential of Duroc × (Landrace × Yorkshire) (DLY), Berkshire × (Duroc × Saba) (BDS), and Duroc × (Berkshire × Saba) (DBS) three-way crossbred pigs were compared. The results showed that DLY had the highest live weight, carcass weight, lean meat percentage, drip loss, glycolysis potential, muscle diameter, and relative mRNA expression levels of type IIb muscle fibers as well as the lowest ultimate pH (*p* < 0.05). The lightness value of DBS was the highest (*p* < 0.05). Among the three crossbred pigs, myristic, arachidic, palmitoleic, and eicosenoic acids were the highest in BDS. These results indicated that the carcass traits of local crossbred pigs were worse than those of DLY pigs, but meat quality was markedly higher, with BDS showing the best meat quality.

## Introduction

With rapid global population and economic growth, meat production and consumption have increased substantially in recent decades (1). More than one third of the world's population consumes pork, making it one of the main sources of animal protein for humans (2). As an important economic trait, meat quality is mainly determined by edibility and nutritional quality and is affected by numerous factors such as variety, muscle characteristics, and production settings (3). Duroc × (Landrace × Yorkshire) (DLY) pigs are popular in the Chinese pig industry because of their fast growth rate, high lean meat percentage, and high meat productivity (4). However, their meat quality does not currently meet consumer demand (5).

As one of the largest pig producers worldwide, China has many indigenous pig breeds with unique genetic characteristics that have developed over thousands of years due to domestication and natural selection (6). Saba pigs, which are produced in high-altitude areas of Yunnan, exhibit excellent characteristics in meat quality, crude feed utilization rate, and reproduction rate (7). However, purebred local pigs are rarely produced commercially in China because of their low feed/gain ratio and lean meat percentage.

Owing to heterosis and breed complementarity, hybrid offspring frequently exhibit better performance than their paternal and maternal lines. Local animal breeds (especially pigs and poultry) are often used in two-way and three-way crossbreeding systems to produce progenies with superior meat quality (6, 8). Three-way terminal crosses are widely used in commercial pig production and show higher production efficiency than pure breeds or two-way crosses (9). Berkshire pigs are renowned for their excellent lean meat quality; thus, crossing them with local pigs can improve carcass traits in the resulting offspring. In addition, Duroc pigs are often used as terminal sires in crossbreeding because of their fast growth rate, high feed conversion efficiency, and lean meat percentage (10).

To date, Saba pigs have not been effectively developed and utilized, and the meat quality of three-way crossbred Saba, Berkshire, and Duroc pigs remains poorly explored. This study aimed to compare and analyze the meat quality, fatty acid and muscle fiber composition, and glycolysis potential in DLY, Berkshire × (Duroc × Saba) (BDS), and Duroc × (Berkshire × Saba) (DBS) three-way crossbred pigs. Our findings provide data for the research, development, and utilization of Saba pig breeds and their genetic resources as well as scientific evidence for pig breeding systems that produce high-quality pork and promote the utilization of local pig breed resources in China.

## Materials and methods

### Animals and sample collection

In total, 100 weaned BDS, DBS, and DLY piglets (35 days old) were selected and raised in the same building (20 pigs per pen), equipped with a fully slatted floor, feeders, and nipple drinkers. All pigs were raised in professional breeding cooperatives in Pude Village, Malutang Township, Luquan County, Kunming City, Yunnan Province, China. The pigs were fed the same National Research Council (2012) three-stage diet (Supplementary Table 1) until 210 days of age. Ten healthy and weight-matched pigs of each breed were randomly selected. Weighed and recorded (live weight) after fasting for 24 h, then euthanized *via* exsanguination. Subsequently, carcass measurements were collected according to Song et al. (11) method (11), including carcass weight (remove head, hoof, tail and viscera), carcass length (the distance from the pubic symphysis leading edge to the fovea of the first cervical spine on the left side of the carcass) and lean meat rate (the percentage of lean meat weight to carcass weight). The longissimus dorsi muscle of each pig was then removed to evaluate meat quality and glycolysis potential. The samples were placed in a refrigerator at 4°C, and meat quality characteristics were evaluated after 45 min. Moreover, a section of each muscle sample was divided, packaged, and frozen at −20°C to measure lactate, glycogen, and pH at 24 and 48 h. All animal experiments were approved by the Institutional Animal Care and Use Committee of Yunnan Agricultural University (No. YNAU20211004).

### Meat quality characteristics

Moisture and lipid contents were measured as per the standards provided by the Association of Official Analytical Collaboration (AOAC) International (12). Drip loss was determined following methods outlined by Choi et al. (13), with slight modifications. Longissimus dorsi muscle samples (2 × 3 × 5 cm) were taken, weighed, and then vacuum-packed in a plastic bag. After refrigeration (4°C) for 24 h, the sample was weighed again, and the weight difference of the sample was considered the drip loss. A tenderness meter (C-LM3B, Harbin, China) was used to measure the shear force of the longissimus dorsi muscle, and each sample was measured three times. A portable pH meter (HANNA HI9125, Italy) was used to measure the pH of the longissimus dorsi muscle. The pH meter was calibrated with standard buffers of pH 7.0 and 4.0 prior to experimental readings. A colorimeter (CR-400, Minolta, Japan) was used to measure the meat color of muscles, and each sample was measured three times.

### Measurements of fatty acids

Total fat was extracted as previously described (14). The fatty acid content of the obtained sample was determined according to the method of Lee et al. under a modified oven temperature (15). Briefly, gas chromatography [TRACE 1310, Jinheng Instrument (Shanghai) Co., Ltd. China] was used to separate and quantify fatty acid methyl ester. The initial oven temperature was set to 100°C, held for 13 min, then increased at a rate of 10°C/min to 180°C, held for 6 min, then again increased at a rate of 1°C/min to 200°C, held for 20 min, and finally increased at a rate of 4°C/min to 230°C and held for 10.5 min. The temperatures of the injector and detector were 270°C and 280°C, respectively.

### Quantitative real-time polymerase chain reaction (qRT-PCR)

The RNAprep Pure Tissue Kit (Tiangen Biochemical Technology Co., Ltd., Beijing, China) was used to extract total RNA, and 3.0% agarose gel electrophoresis was used to detect the concentration and purity of RNA samples. First-strand cDNA was then synthesized using the FastKing RT Kit (with gDNase) (Tiangen Biochemical Technology Co., Ltd., Beijing, China). PCR primers are shown in Supplementary Table 2. The reaction system contained template cDNA (2 μL), ddH2O (6.8 μL), forward primer (0.6 μL), reverse primer (0.6 μL), and 2 × SuperReal PreMix Plus (10.0 μL). PCR was conducted under the following conditions: initial single cycle denaturation at 95°C for 30 s, followed by 40 cycles of 95°C for 5 s, and finally at 60°C for 30 s. The 2-ΔΔCt method was used to calculate relative gene expression.

### Histochemical characteristics

The meat samples were sliced (10 μm) using a low-temperature microtome at −20°C (SYD-K2040, Shenyang Yude Electronic Instrument Co., Ltd., China). Then, using the improved Brooke and Kaiser (16) method, samples were incubated for histochemical demonstration of myosin adenosine triphosphatase after pre-incubation under alkaline (pH 10.4) and acidic (pH 4.35) conditions. The stained sections were examined using an image analysis system (Nikon E600, Nikon Corporation, Japan). Muscle fibers were classified into types I, IIa, and IIb according to Brooke and Kaiser (16). Note that types IIA and IIB could not be clearly defined using the alkali method. Image-Pro Plus (v6.0) was used to measure the fiber diameter of each sample.

### Glycolytic metabolite measurement

Frozen samples were pulverized, and glycogen and lactate concentrations were measured according to the method reported by Luo et al. (17). Glycolytic potential (GP) (μmol/g meat) was determined according to the formula of Li et al. (18): GP = 2 × (glycogen) + (lactic acid).

### Statistical analysis

SPSS (v21) was used for one-way ANOVA. Multiple comparisons were performed using Duncan's multiple range test. At *p* < 0.05, data were statistically significant, and results are presented as mean ± standard error of the mean (SEM).

## Results

### Carcass characteristics

Comparisons of the longissimus dorsi muscle and carcass characteristics among DLY, BDS, and DBS are shown in Table 1. In DLY, live weight and carcass weight were significantly higher than those in BDS and DBS, and the lean meat rate was significantly higher than that in DBS (*p* < 0.05). Carcass weight in DBS was significantly higher than that in BDS (*p* < 0.05). However, there was no significant difference in carcass length among the three pig breeds.

**Table 1:** Comparison of carcass characteristics among DLY, BDS, and DBS.

**Items**	**DLY**	**BDS**	**DBS**
Live weight (kg)	128.20 ± 2.33^a^	105.15 ± 2.54^b^	113.90 ± 3.75^b^
Carcass weight (kg)	99.92 ± 1.76^a^	73.50 ± 2.03^c^	90.78 ± 3.59^b^
Carcass length (cm)	88.17 ± 3.34	85.51 ± 1.19	91.20 ± 1.58
Lean meat rate (%)	61.61 ± 0.25^a^	58.27 ± 4.17^ab^	52.31 ± 2.68^b^

In the same row, significant differences (p < 0.05) are represented as different superscript letters. DLY, Duroc × (Landrace × Yorkshire); BDS, Berkshire × (Duroc × Saba); DBS, Duroc × (Berkshire × Saba).

### Meat quality characteristics

Table 2 summarizes the meat quality characteristics of the longissimus dorsi muscle for DLY, BDS, and DBS. No significant differences in water and fat content were found among DLY, BDS, and DBS. Regarding meat quality characteristics, the drip loss of DLY was highest, with a significantly higher value than that of BDS and DBS (*p* < 0.05). Over time, pork pH showed a downward trend in all three breeds. At 45 min, the pH was significantly higher in DLY and BDS than in DBS (*p* < 0.05). At 24 h, the pH was highest in BDS and significantly higher than in DLY (*p* < 0.05). At 48 h, the pH was lowest in DLY (*p* < 0.05). No significant differences were found among the three pig breeds in terms of the shear force. For longissimus dorsi muscle color, BDS showed the lowest lightness value; the lightness of BDS was significantly lower than that of DBS (*p* < 0.05), while redness and yellowness were not significantly different among the three breeds.

**Table 2:** Comparison of meat quality characteristics among DLY, BDS, and DBS.

**Item**	**DLY**	**BDS**	**DBS**
Moisture (%)	68.57 ± 1.22	67.84 ± 0.25	67.45 ± 0.41
Fat (%)	3.92 ± 0.53	3.88 ± 0.32	4.18 ± 0.30
Drip loss (%)	2.82 ± 0.31^a^	1.53 ± 0.18^b^	2.15 ± 0. 17^b^
pH (45 min)	6.40 ± 0.13^a^	6.31 ± 0.08^a^	5.88 ± 0.05^b^
pH (24 h)	5.54 ± 0.05^b^	5.68 ± 0.03^a^	5.62 ± 0.03^ab^
pH (48 h)	5.41 ± 0.01^b^	5.52 ± 0.02^a^	5.57 ± 0.03^a^
Shear force (kg/cm^2^)	5.08 ± 0.37	5.91 ± 0.83	5.48 ± 0.48
**Meat color**			
Lightness	41.56 ± 1.29^ab^	39.21 ± 1.11^b^	42.84 ± 0.73^a^
Redness	6.81 ± 1.01	6.36 ± 0.33	6.92 ± 0.41
Yellowness	1.63 ± 0.52	1.32 ± 0.09	1.59 ± 0.25

In the same row, significant differences (p < 0.05) are represented as different superscript letters. DLY, Duroc × (Landrace × Yorkshire); BDS, Berkshire × (Duroc × Saba); DBS, Duroc × (Berkshire × Saba).

### Fatty acid analysis

The fatty acid composition in the longissimus dorsi muscles of the DLY, BDS, and DBS groups are compared in Table 3. Oleic, palmitic, linoleic, and stearic acid are the main fatty acids in longissimus dorsi muscle. Compared to DLY and DBS, BDS had higher concentrations of myristic, arachidic, palmitoleic, and eicosenoic acid (*p* < 0.05). Other fatty acid levels were not significantly different among the three breeds.

**Table 3:** Relative percentages of fatty acids in longissimus dorsi muscle from DLY, BDS and DBS.

**Fatty acid (%)**	**DLY**	**BDS**	**DBS**
Capric acid (C10:0)	0.1138 ± 0.009	0.19 ± 0.037	0.122 ± 0.017
Lauric acid (C12:0)	0.10 ± 0.00458	0.16 ± 0.0396	0.10 ± 0.0175
Myristic acid (C14:0)	1.39 ± 0.073^b^	2.73 ± 0.69^a^	1.46 ± 0.197^b^
Pentadecanoic acid (C15:0)	0.037 ± 0.002	0.053 ± 0.01	0.027 ± 0.005
Palmitic acid (C16:0)	24.55 ± 0.44	31.09 ± 5.34	26.88 ± 4.00
Heptadecanoic acid (C17:0)	0.19 ± 0.02	0.27 ± 0.07	0.14 ± 0.02
Stearic acid (C18:0)	11.88 ± 0.54	19.52 ± 6.56	13.40 ± 2.36
Arachidic acid (C20:0)	0.19 ± 0.005^b^	0.33 ± 0.07^a^	0.20 ± 0.0357^b^
Myristoleic acid (C14:1)	0.022 ± 0.004	0.044 ± 0.012	0.0298 ± 0.006
Palmitoleic acid (C16:1)	2.93 ± 0.23^b^	5.27 ± 1.12^a^	2.96 ± 0.40^b^
Elaidic acid (C18:1n9t)	0.17 ± 0.02	0.22 ± 0.05	0.14 ± 0.03
Oleic acid (C18:1n9c)	42.95 ± 0.48	28.63 ± 8.54	33.54 ± 6.62
Linoleic acid (C18:2n6)	12.22 ± 0.68	6.99 ± 2.13	18.09 ± 8.30
γ-Linoleic acid (C18:3n6)	0.049 ± 0.009	0.037 ± 0.011	0.033 ± 0.0025
Linolenic acid (C18:3n3)	0.45 ± 0.04	0.57 ± 0.16	0.37 ± 0.04
Eicosenoic acid (C20:1)	0.70 ± 0.04^b^	1.42 ± 0.30^a^	0.83 ± 0.15^b^
Cis-11,14-Eicosadienoic acid (C20:2)	0.43 ± 0.04	0.62 ± 0.17	0.39 ± 0.07
Cis-8,11,14-Eicosatrienoic acid (C20:3n6)	0.21 ± 0.03	0.25 ± 0.05	0.16 ± 0.02
Cis-11,14,17-Eicosatrienoic acid (C20:3n3)	0.074 ± 0.006	0.107 ± 0.039	0.076 ± 0.02
Arachidonic acid (C20:4n6)	1.18 ± 0.31	1.34 ± 0.25	0.96 ± 0.14
cis-5,8,11,14,17-Eicosapentaenoic acid (C20:5n3)	0.07 ± 0.01	0.06 ± 0.01	0.06 ± 0.01
Cis-4,7,10,13,16,19-Docosahexaenoic acid (C22:6n3)	0.14 ± 0.01	0.18 ± 0.04	0.10 ± 0.01
Saturated fatty acid (SFA)	38.45 ± 0.79	54.36 ± 6.15	42.32 ± 6.60
Unsaturated fatty acid (USFA)	61.55 ± 0.79	45.64 ± 6.15	57.68 ± 6.60
Mono-USFA	46.76 ± 0.67	35.58 ± 7.49	37.49 ± 6.67
Poly-USFA	14.80 ± 0.81	10.06 ± 1.97	20.19 ± 8.18
USFA/SFA	1.61 ± 0.06	0.97 ± 0.23	1.74 ± 0.54

In the same row, significant differences (p < 0.05) are represented as different superscript letters. DLY, Duroc × (Landrace × Yorkshire); BDS, Berkshire × (Duroc × Saba); DBS, Duroc × (Berkshire × Saba).

### Muscle fiber diameter and muscle myosin heavy chain (MyHC) expression

**Figure 1:**
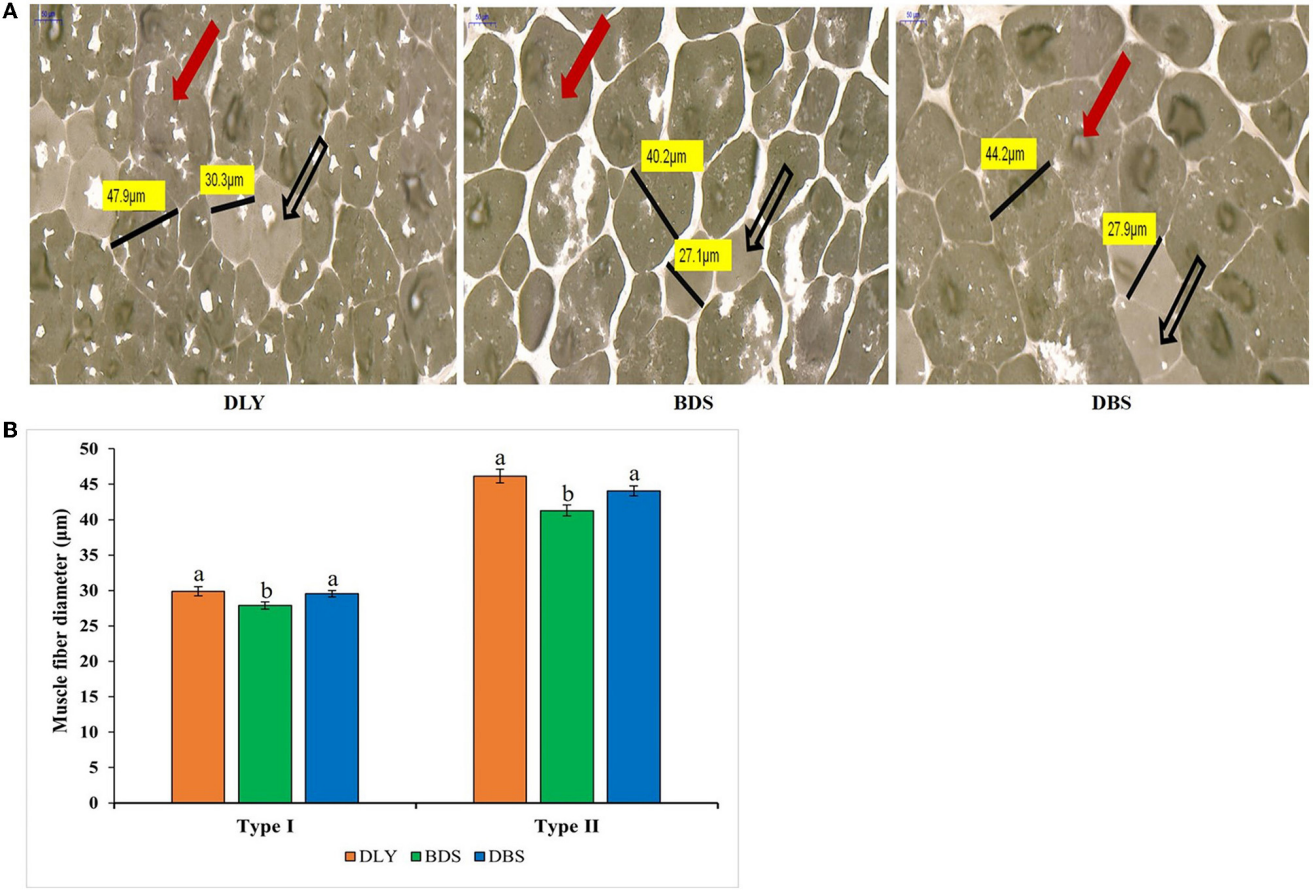
Muscle fiber typing and diameter measurement. **(A)** Frozen sections of ATPase in longissimus dorsi muscle of different hybrid pigs. Black hollow arrow indicates type I muscle fiber, red solid arrow indicates type II muscle fiber, and black line segment is measured length of muscle fiber diameter. **(B)** Comparison of longissimus dorsi muscle fiber diameter from different hybrid pigs. Significant differences (*p* < 0.05) are indicated by different letters. DLY, Duroc × (Landrace × Yorkshire); BDS, Berkshire × (Duroc × Saba); DBS, Duroc × (Berkshire × Saba).

Using ATPase staining, we compared the diameters of the longissimus dorsi muscle fibers of the three breeds. As shown in Figure 1, the diameters of the type I and II muscle fibers were highest in DLY, and were significantly higher in DLY and DBS than in BDS (*p* < 0.05).

**Figure 2:**
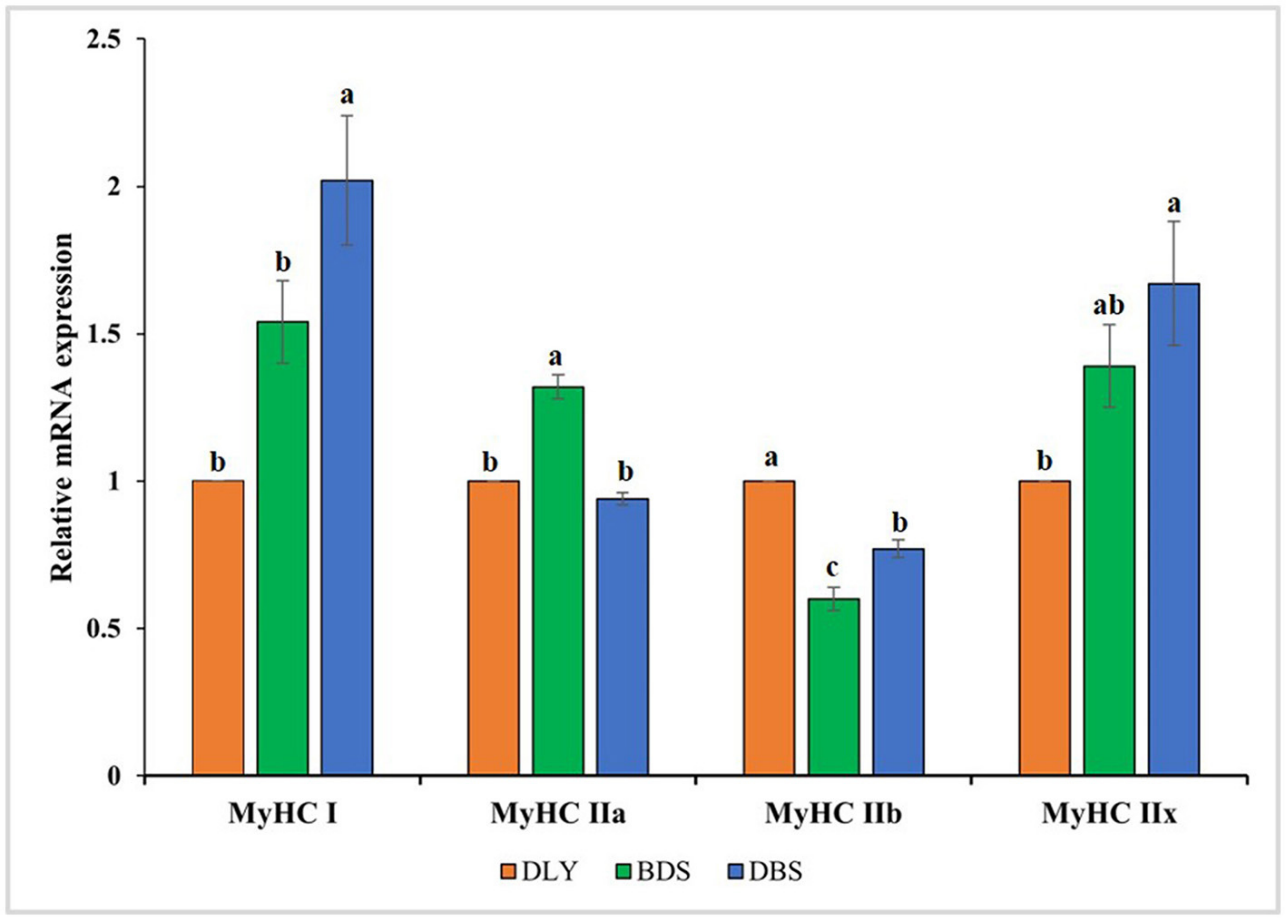
Relative mRNA expression of longissimus dorsi muscle myosin heavy chain (MyHC) isoforms in DLY, BDS and DBS. Different superscript letters indicate significant differences (*p* < 0.05).

The relative mRNA expression levels of myosin heavy chain (MyHC) in DLY, BDS, and DBS were measured using qRT-PCR (Figure 2). Four MyHC isoforms were detected in the longissimus dorsi muscle of three pig breeds: MyHC I, MyHC IIa, MyHC IIb, and MyHC IIx. The results showed that MyHC I had the highest expression in DBS, significantly higher than in BDS and DLY (*p* < 0.05), and MyHC IIx was significantly higher in DBS than in DLY (*p* < 0.05). Furthermore, BDS had the highest expression of MyHC IIa but the lowest expression of MyHC IIb compared to DBS and DLY (*p* < 0.05). DLY showed the highest MyHC IIb expression (*p* < 0.05).

### Glycolytic potential

The longissimus dorsi muscle GP in the three hybrid pig breeds is shown in Table 4. Glycogen content was the lowest in BDS and significantly lower than that in DLY (*p* < 0.05). Compared with BDS and DBS, DLY had the highest lactate content and GP value (*p* < 0.05).

**Table 4:** Comparison of GP in longissimus dorsi muscles of DLY, BDS, and DBS.

**Items**	**DLY**	**BDS**	**DBS**
Glycogen (μmol/g)	1.17 ± 0.03^a^	0.84 ± 0.09^b^	0.99 ± 0.03^ab^
Lactate (μmol/g)	129.35 ± 1.70^a^	110.55 ± 4.10^b^	114.96 ± 0.14^b^
GP (μmol/g)	131.68 ± 1.69^a^	112.24 ± 4.20^b^	116.94 ± 0.09^b^

In the same row, significant differences (p < 0.05) are represented as different superscript letters. DLY, Duroc × (Landrace × Yorkshire); BDS, Berkshire × (Duroc × Saba); DBS, Duroc × (Berkshire × Saba).

## Discussion

Pork quality is an important economic characteristic that affects consumers' purchase decision (19). Crossbreeding with local pigs is important for the production of high-quality pork (20), but carcass traits in crossbred pigs may decline because of the slow growth of local breeds. For example, Jiang et al. (21) found that the carcass traits of Chinese Landrace × Meishan and Duroc × Landrace × Meishan pigs were significantly lower than those of foreign crossbred pigs (*p* < 0.05). This is consistent with our research, showing lower live weight and carcass weight in Chinese local hybrid pigs (BDS and DBS) than in foreign hybrid pigs (DLY). Our results also showed that BDS had a higher lean meat percentage and lower live weight, carcass weight, and carcass length than DBS, indicating differences in carcass traits of pig breeds obtained by different crossbreeding methods.

Studies have reported obvious differences in meat quality between hybrids (22). Water is the main component of meat and an essential parameter for evaluating meat quality; loss of water in meat can lead to a decrease in pH, and the meat with higher drip loss is more acid and lighter (23). We detected the highest drip loss and lowest pH in DLY pigs. Ultimate pH is a primary factor affecting meat quality in the early postmortem stage, and protein denaturation and drip loss increase as muscle pH decreases (24). Meat color is an important trait affecting consumer purchasing behavior. Cameron et al. (25) showed that color lightness is negatively correlated with meat tenderness, flavor, and acceptability. Kim et al. (26) found that DLY pigs had higher lightness than Kagoshima Berkshire, British Berkshire (Yorkshire × Berkshire) × Berkshire, and Korean native black pig × wild boar pigs, indicating that lightness values differ in different hybrid pigs. In our study, we found that BDS had the lowest lightness values and were significantly lower than those of DBS. Thus, BDS meat quality was better than that of DLY meat in terms of drip loss, pH, and lightness, and better than DBS meat in terms of lightness.

Fatty acid composition is one of the main contributors to pork flavor and is influenced by breed and genotype (27). Highly saturated fatty acids (SFAs) are considered to have positive effects on stabilizing fat oxidation (28). Cameron et al. (27) also found that palmitoleic acid was positively correlated with pork flavor and overall acceptability. In this study, compared to DLY and DBS, BDS had the highest SFA (myristic acid and arachidic acid) and palmitoleic acid content and the best meat quality traits (drip loss, pH, and lightness), similar to earlier studies. In terms of fatty acid content for human health, long-chain mono-unsaturated fatty acids (USFAs) such as C20:1 can ameliorate obesity-related metabolic dysfunction and promote health (29). In our study, BDS contained a higher proportion of C20:1, suggesting that BDS may produce meat that is more beneficial to human health than that of DLY and DBS. Thus, BDS was superior to DLY and DBS in terms of overall meat quality.

Muscle fiber characteristics are key factors affecting pork quality (30). Miao et al. (31) reported that Luchuan pork is superior to Duroc pork in part due to its smaller muscle fiber diameter and area. Similarly, commercial broilers with larger muscle fiber diameters have poorer meat tenderness than native and hybrid chickens in China (32). In this study, muscle fiber diameter was significantly lower in BDS pigs than in DLY and DBS pigs (*p* < 0.05), suggesting better meat quality. Pig muscle fibers can be categorized into four myosin isoforms: MyHC I, MyHC IIa, MyHC IIb, and MyHC IIx. It is generally believed that meat with a higher abundance of type I and type IIa fibers are of higher quality with improved oxidation ability compared to that of meat rich in type IIb fibers (33). Type II muscle fibers grow faster, and their content is related to higher body weight; however, rapidly increasing body weight can affect the paleness and exudative properties of pork (34). Huang et al. (35) showed that the relative mRNA expression level of MyHC IIb was highest in Landrace pigs at 60 days of age, while the relative mRNA expression levels of MyHC I and IIa were highest in local pigs. Similarly, in our study, the highest relative mRNA expression of MyHC in DBS and BDS of local crossbred pigs was type I and type IIa, respectively, while that of commercial crossbred pigs in DLY was type IIb, which may be an important factor regarding the meat quality of local hybrid pigs is superior to that of commercial pigs.

The storage of muscle glycogen during slaughter and the rate of glycolysis after slaughter can affect meat quality by affecting the rate and degree of postmortem pH decline (36). Glycogen, lactate, and GP levels are negatively correlated with ultimate pH but positively correlated with drip loss (37). In addition, Schilling et al. showed that a high level of glycolysis potential was significantly related to the decrease of meat quality, such as color and drip loss (38). Przybylski et al. (23) found that chicken with deeper glycolysis and higher drip loss produced more methylglyoxal, which may change the protein properties of muscle (23). Previous research has also shown that Chinese pure native and hybrid pigs have a lower GP and better meat quality than commercial DLY pigs (17, 39), consistent with our findings. The glycogen, lactate, and GP levels in the longissimus dorsi muscles increased in the order of BDS < DBS < DLY. Correspondingly, meat quality was the best for BDS and the worst for DLY.

## Conclusions

In this study, live weight, carcass weight, and lean meat percentage were higher in DLY than in BDS and DBS. However, drip loss, glycogen content, lactate content, GP level, and ultimate pH level were lower in BDS and DBS than in DLY. In addition, saturated fatty acid (myristic acid and arachidic acid) and monounsaturated fatty acid (palmitoleic acid and eicosenoic acid) contents were higher in BDS than in DLY and DBS. Relative mRNA expression of type I and type IIa muscle fibers, which are beneficial to meat quality, were highest in DBS and BDS, respectively (*p* < 0.05), while the relative mRNA expression of type IIb muscle fibers, which are negatively related to meat quality, was highest in DLY (*p* < 0.05). Thus, compared with commercial DLY pigs, Chinese local hybrid BDS and DBS pigs had poorer carcass traits, but better meat quality, while BDS had the best meat quality and flavor. These findings provide basic data for promoting the conservation and utilization of Saba pigs and the production of high-quality pork in the pig industry.

## Data availability statement

The original contributions presented in the study are included in the article/Supplementary material, further inquiries can be directed to the corresponding authors.

## Ethics statement

The animal study was reviewed and approved by the Institutional Animal Care and Use Committee of Yunnan Agricultural University. Written informed consent was obtained from the owners for the participation of their animals in this study.

## Author contributions

HH and HP designed the experiments and revised this manuscript. JR, HJ, XiL, XuL, YP, YiH, SZ, and CS performed the experiments. YL and YaH analyzed the data and wrote the manuscript. All the authors contributed to the article and approved the submitted version.
