# Supplementary material for: Comparison of meat quality and glycolysis potential of two hybrid pigs in three-way hybrid model
Source: Front Vet Sci. 2023 Feb 17;10:1136485. doi: 10.3389/fvets.2023.1136485 (PMC9981941; doi:10.3389/fvets.2023.1136485)
Supplement: Supplementary file 1 [file Data_Sheet_1.docx]

**Supplementary table 1.** Basic dietary composition and nutritional levels

|  | Piglet  (5–10 kg) | Growing pig  (20–60 kg) | Fattening pig  (60 kg–210 day) |
| --- | --- | --- | --- |
| **Ingredients** | | | |
| Corn | 49.5 | 49.9 | 53.2 |
| Soybean meal | 23.5 | 15 | 14 |
| Wheat bran | 15 | 22 | 18 |
| Alfalfa meal | 5 | 8 | 9 |
| CaHPO_4_ | 1.8 | 0.6 | 0.6 |
| Lysine | 0.9 | 0.1 | 0.5 |
| L-Methionine | 0.5 | 0.4 | 0.4 |
| Soya bean oil | 2.6 | 2.5 | 2.8 |
| NaCl | 0. 2 | 0.5 | 0.5 |
| Premix^1^ | 1^1)^ | 1^2)^ | 1^3)^ |
| Total | 100 | 100 | 100 |
| **Nutrient levels** | | | |
| Digestible energy (MJ/kg) | 3 251.04 | 3 115.65 | 3 152.55 |
| Crude protein | 19.614 | 16.278 | 15.965 |
| Crude fiber | 3.842 | 5.336 | 5.422 |
| Ca | 0.742 | 0.454 | 0.456 |
| Total P | 0.82 | 0.581 | 0.568 |
| Lysine | 1.185 | 0.888 | 1.137 |
| Methionine + Cystine | 0.495 | 0.396 | 0.396 |

^1^ Premix supplied per kg diet: FeSO_4_, 748 mg; ZnSO_4_, 477 mg; CuSO_4_, 26 mg; MnSO_4_, 12.8 mg; KIO_3_, 0.26 mg; Na_2_SeO_3_, 0.57 mg; vitamin A, 2 276 IU; vitamin D, 228 IU; vitamin E, 11 IU; vitamin K, 2.2 mg; vitamin B1, 1.3 mg; vitamin B2, 3.1 mg; vitamin B3, 23 mg; vitamin B5, 13.4 mg; vitamin B7, 0.11 mg; vitamin B9, 0.68 mg; vitamin B12, 23 μg.

^2^ Premix supplied per kg diet: FeSO_4_, 564 mg; ZnSO_4_, 505 mg; CuSO_4_, 19 mg; MnSO_4_, 6.8 mg; KIO_3_, 0.24 mg; Na_2_SeO_3_, 1.0 mg; vitamin A, 1 230 IU; vitamin D, 189 IU; vitamin E, 10 IU; vitamin K, 2.5 mg; vitamin B1, 1.0 mg; vitamin B2, 2.5 mg; vitamin B3, 13 mg; vitamin B5, 10 mg; vitamin B7, 0.09 mg; vitamin B9, 0.57 mg; vitamin B12, 10 μg.

^3^ Premix supplied per kg diet: FeSO_4_, 62 mg; ZnSO_4_, 413 mg; CuSO_4_, 16 mg; MnSO_4_, 7.8 mg; KIO_3_, 0.24 mg; Na_2_SeO_3_, 0.96 mg; vitamin A, 1 225 IU; vitamin D, 118 IU; vitamin E, 10 IU; vitamin K, 2.1 mg; vitamin B1, 1.0 mg; vitamin B2, 2.1 mg; vitamin B3, 9 mg; vitamin B5, 10 mg; vitamin B7, 0.09 mg; vitamin B9, 0.57 mg; vitamin B12, 10 μg.

**Supplementary table 2.** Primer sequences of PCR

| Gene | Primer sequences (5'→3') |
| --- | --- |
| MyHC I | F: CGACACACCTGTTGAGAAG  R: AGATGCGGATGCCCTCCA |
| MyHC IIa | F: GGGCTCAAACTGGTGAAGC  R: AGATGCGGATGCCCTCCA |
| MyHC IIb | F: CTTCACTGGCGCAGCAGGT  R: AGATGCGGATGCCCTCCA |
| MyHC IIx | F: GTTCTGAAGAGGGTGGTAC  R: AGATGCGGATGCCCTCCA |
| ACTB | F: TTTCTTCTGCCATTTTCCT  R: ATACACCCACAGCACCTT |
